# Prediction of response to thrombolysis in acute stroke using neural network analysis of CT perfusion imaging

**DOI:** 10.1177/23969873231183206

**Published:** 2023-06-23

**Authors:** Yutong Chen, Daniel J Tozer, Weiran Liu, Edward J Peake, Hugh S Markus

**Affiliations:** 1 Stroke Research Group, Department of Clinical Neurosciences, University of Cambridge, Cambridge, UK; 2 Department of Radiology, Cambridge University Hospitals NHS Foundation Trust, Cambridge, UK

**Keywords:** Machine learning, neural network, CT perfusion, thrombolysis

## Abstract

**Background:**

In ischaemic stroke patients undergoing reperfusion therapy, the amount of salvageable tissue, that is, extent of the ischaemic penumbra, predicts the clinical outcomes. CT perfusion (CTP) enables quantification of penumbral tissues to guide decision making, and current programmes have automated its analysis. More advanced machine learning techniques utilising the CTP maps may improve prediction beyond the ischaemic volume measures.

**Method:**

We determined whether applying convolutional neural networks (CNN), a key machine learning technique in modelling image-label relationships, to post-processed CTP maps improved prediction of outcome, assessed by 3 months modified Rankin scale (mRS). Patients who underwent thrombolysis but not thrombectomy were included. CTP maps of a retrospective cohort of 230 patients with middle cerebral artery stroke were used to develop the model, which was validated in an independent cohort of 129 patients.

**Results:**

We constructed a CNN model that predicted a favourable post-thrombolysis outcome (mRS 0–2 at 3 months) with an area under receiver-operator characteristics curve (AUC) of 0.792 (95% CI, 0.707–0.877). This model outperformed a currently clinically used MISTAR software using previously validated thresholds (AUC = 0.583, 95% CI, 0.480–0.686) and a model modified using thresholds from the derivation cohort (AUC = 0.670, 95% CI, 0.571–0.769). By combining CNN-derived features and baseline demographic features, the prediction AUC was improved to 0.865 (95% CI, 0.794–0.936).

**Conclusion:**

CNN improved prediction of post-thrombolysis outcome, and may be useful in selecting which patients benefit from thrombolysis.

## Introduction

Intravenous thrombolysis with recombinant tissue plasminogen activator (rtPA) given within 4.5 h of stroke onset improves functional outcome of acute ischaemic stroke.^1^ More recently thrombectomy has been shown to have a superior outcome in patients with large cerebral artery occlusion.^2^ It has been known for many years that the degree of salvageable tissue in different individuals at the same time points following acute ischaemic stroke varies widely, with the extent of collateral blood supply believed to be an important determinant factor.^3,4^ Recent trials of both intravenous thrombolysis (WAKE-UP^5^), and thrombectomy (DAWN^6^ and DEFUSE 3^7^), have shown benefit outside conventional time windows, and up to 24 h, in those patients who have salvageable or penumbral tissue as identified using advanced imaging techniques. This has led to the suggestion that we should be moving towards a tissue based selection of patients for interventions, rather than using current rigid time cut-offs.^8^

The extent of salvageable tissue can be assessed using MRI,^5^ but acute MRI is not available in many stroke centres.^9^ CT perfusion is more widely available, can be performed rapidly as part of the initial CT brain scan, and is well tolerated by patients. It has been demonstrated to be comparable to MRI for imaging the ischaemic core and penumbra.^10,11^ To assist in rapid treatment decision making in thrombolysis, automated software has been developed to analyse CTP scans to allow easier interpretation by physicians.

Machine learning offers a powerful approach to rapidly and automatically analyse complex multidimensional data such as CTP. For example, decision tree – an early machine learning technique – obtains the optimal cut-off thresholds from the training samples and uses those thresholds to classify inputs into different outputs.^12^ Such an approach has been used to develop predictive models using CTP data in acute stroke,^13,14^ which are now clinically available.^13^ However, these existing techniques extracted core and penumbra volumes from raw image data and then used these as inputs to the machine learning models. These volume measures may miss other subtle image features either within or outside the ischaemic region.^15^ This limitation can be addressed by a machine learning model that associates the CTP maps to the corresponding post-thrombolysis outcomes. This can be achieved by convolutional neural networks (CNN). CNN acts as a feature extractor^16^ and may identify predictive imaging features beyond the core and penumbra.

In this study we investigated whether a CNN model improves the prediction of outcome in acute stroke patients treated with thrombolysis. We developed a CNN algorithm, in a derivation cohort and tested it in a second replication cohort. We compared its performance with a clinically available decision tree method.

## Method

### Study design

The study was designed to compare the performance of a CNN-based analysis of CTP maps with a standard clinically available algorithm to predict functional outcome in patients undergoing thrombolysis. It was a retrospective analysis of a series of consecutive acute stroke patients undergoing CTP for acute middle cerebral artery ischaemic stroke in a single comprehensive stroke centre. In the centre, in October 2009, CTP was implemented as part of the acute imaging pathway of patients being considered for thrombolysis. Acute imaging comprised a non-contrast CT (NCCT) and CTP on admission, and a NCCT 24 h after thrombolysis. Decisions regarding thrombolysis were made based on current clinical guidelines, and CTP was used for diagnostic and research purposes only.

The study included both a derivation cohort – on which our machine learning models were trained – consisting of all eligible patients between November 2012 and May 2017 (*N* = 230), and a replication cohort consisting of all eligible patients presenting between June 2017 and December 2020 (*N* = 129).

### Inclusion and exclusion criteria

Inclusion criteria for both the derivation and replication cohorts were: age >18 years, non-lacunar middle cerebral artery (MCA) stroke, and completed intravenous thrombolysis with rtPA. Exclusion criteria were: non-MCA strokes (suspected or proven posterior circulation, anterior cerebral artery strokes, lacunar strokes), previous stroke, bilateral stroke, acute endovascular therapy performed (intra-arterial thrombolysis or mechanical thrombectomy), 3 months mRS not available, CTP data unavailable or un-processable. Thrombolysis outside the 4.5 h window was not a part of the exclusion criteria.

**Figure 1.**
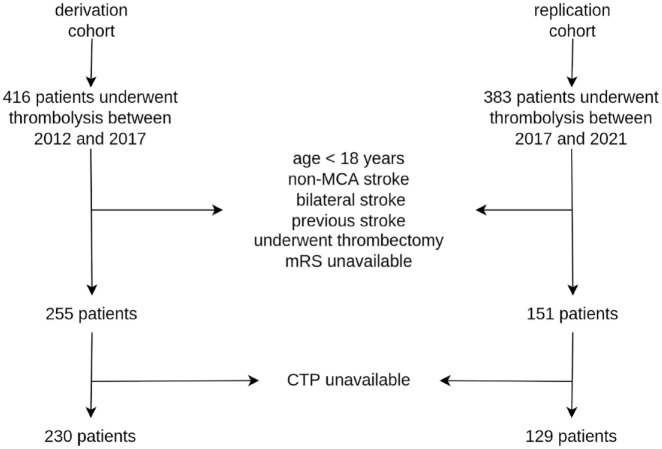
Patient selection flow chart. The derivation cohort consists of both the training and validation datasets.

To compile the derivation cohort, we identified a total of 416 patients who underwent thrombolysis therapy between November 2012 and May 2017, of which 230 met the inclusion criteria. For the replication cohort, we identified 383 patients who underwent thrombolysis between June 2017 and December 2021, of which 129 patients met the inclusion criteria (Figure 1). No stratifications were performed on the derivation and the replication cohorts with respect to mRS at 3 months.

Ethical approval was gained from the Cambridge University Hospitals Trust research ethics committee (Project IRAS ID: 244503).

### Assessment of patient characteristics

The following clinical characteristics were recorded from the medical notes; age, sex, vascular risk factors (hypertension, diabetes, hypercholesterolaemia, smoking, atrial fibrillation, ischaemic heart disease), admission National Institute of Health Stroke Scale (NIHSS) and delay from symptom onset to thrombolysis. Patients are routinely followed up in clinics 3 months after stroke. The mRS^17^ was estimated from the descriptions of the patients’ post-stroke recovery in clinic follow-up letters as a measure of stroke outcome, as this has been widely adopted in the thrombolysis trials as a reliable outcome measure. Separate researchers carried out data collection and analysis (W.L. and Y.C. respectively) to ensure the analyst was blinded to the patient features and post-thrombolysis outcome.

### CTP analysis

We compared our CNN approach with a clinically available decision tree algorithm implemented using MISTAR (Apollo Medical Imaging Technology, Melbourne, Australia) software.^18^ The software automatically calculates the hypoperfusion, core and penumbra volumes for pre-defined perfusion parameters and thresholds. We used the default manufacture parameters: hypoperfusion lesion – region with a relative delay time (DT) >3 s; ischaemic core – region within the hypoperfusion lesion with a relative cerebral blood flow (CBF) <30%; penumbra – the difference between the hypoperfusion lesion and the core lesion. These parameters have been validated by a series of previous studies using large datasets^19^ to estimate core and penumbra volumes.

Two MISTAR models were tested compared with the CNN model: 1) MISTAR (original): this utilised the parameters in the original study,^13^ 2) MISTAR (modified): the derivation cohort was used to derive a new set of thresholds. The derived thresholds were 10.8 mL for core volume, 72.0 mL for penumbra volume, and 29.4 mL for volume with delay time >6 s (DT6). For comparison, in the original study, the thresholds were 25 mL for core volume, 20 mL for penumbra volume and 30 mL for DT6 volume.^13^

#### Data preprocessing

CTP scans were acquired by SIEMENS SOMATOM Definition scanners, which processed the raw perfusion signals to generate perfusion maps. For each CTP scan, between 16 and 27 CT volumes were acquired. Each volume was 512 × 512 × [22–32] voxels in size. The in-plane resolutions of the images were 0.420 mm (±0.035 mm). The resolutions between slices were 3.22 mm (±0.415 mm). The average time interval between two consecutive CT volumes was 1.86 s (±0.017 s).

The CTP scans were anonymised and downloaded from the Addenbrookes Picture Archiving and Communication System (PACS) using an in-house automated pipeline. The images were stored as a series of 2D slices as Dicom files. Overlays, including heatmap colour bars showing the colour scale on CTP maps, were removed. Secondary captures were removed from the dataset, and a sub-sample of studies were checked to ensure all burnt-in patient identifiable information had been removed.

On the CTP maps, voxel value was rescaled between 0 and 1 per 3D volume per perfusion map. In each patient, 12–14 axial slices were selected. To reduce storage space, increase computation speed and model performance (Supplemental Table 1), each slice was downsampled to a resolution of 128×128 pixels. All slices from a patient were concatenated and stored as a single NIfTI file. All image processing steps were carried out in python 3.8.11 on Ubuntu 18.04.

#### Statistics

To investigate which clinical and CTP features predicted outcome, Spearman correlation was calculated between each feature and 3 months mRS. Spearman correlation was used instead of Pearson correlation, because none of the features were normally distributed using Shapiro and Wilk test.^20^ Bonferroni correction was applied for multiple comparisons. Correlation analysis was carried out using the cor.test function from stats package in R.

Prediction performance of the CNN was assessed by area under the curve (AUC), accuracy, sensitivity and specificity of identifying favourable (mRS 0–2) and unfavourable outcomes (mRS 3–6). AUC was calculated using the auc function from the pROC package in R.^21^ Using the estimated confidence interval,^22^ comparison of AUC from different methods was made using unpaired *t* test.

To leverage the imaging processing functions of CNN and the interpretability of machine learning models such as lasso and support vector machine (SVM), we tested whether combining CNN-derived features with baseline clinical features would further improve prediction accuracy. To combine the imaging and clinical attributes, we extracted the output of the global average pooling layer of the CNN model. This output represents features extracted by CNN that have predictive values of post-stroke mRS. We then combined these CNN-derived features with clinical features in various machine learning models as described in the Machine learning section.

#### Machine learning

The derivation cohort was split into training and validation sets in a 8:2 ratio using createDataPartition function in caret package in R.^23^ The training set contains 184 samples while the validation set 46. Categorical data such as sex, smoking, and hypertension status was coded as 0 and 1. We defined the following features as baseline demographic features: age, sex, vascular risk factors (hypertension, diabetes, hypercholesterolaemia, smoking, atrial fibrillation, ischaemic heart disease), admission NIHSS and admission mRS. In the derivation cohort, admission NIHSS and mRS were missing in 0.87% of the patients. In the replication cohort, diabetes, hypercholesterolaemia, heart failure status and admission NIHSS were missing in 0.78%, 1.56%, 0.78% and 3.88% of the patients respectively. The missing data was imputed by k-nearest neighbours (KNN). Three-month mRS was not included into the KNN model for imputation.

Machine learning models were built using the caret package. The following clinical features were used as inputs to the machine learning models: age, sex, hypertension status, previous ischaemic heart disease, history of heart failure, status of hypercholesterolaemia, smoking history, diabetes, pre-stroke mRS, and baseline NIHSS. Each variable was centred and scaled. Variables that correlated with each other with coefficient more than 0.9 were removed. We tested the following models: KNN,^24^ SVM^25^ with linear or radial kernels, random forest (RF),^26^ least absolute shrinkage and selection operator (lasso),^27^ and Gaussian Process with radial kernel (GPR).^28^ Prediction accuracy using five-fold cross validation was used to select optimal hyperparameters of each model. The accuracy of each model on the testing dataset was calculated using the confusionMatrix function from the caret package. All machine learning analysis was carried in R version 4.1 on Ubuntu 22.04.

#### Convolutional neural network

Convolutional neural network (CNN) was constructed using pytorch package.^29^ The input consisted of the cerebral blood flow, cerebral blood volume and the time-to-peak maps. The CNN consists of five 3-dimensional convolution layers, followed by an efficient channel attention (ECA) layer to attend to the most important features in the convolutional channels.^30^ This is followed by a global average pooling layer, a dense connection layer of 256 units, and a sigmoid activation layer. The final output is predicted mRS at 3 months.

To augment the training dataset, in every training epoch, the CTP maps were randomly flipped vertically, horizontally or along the z axis. Each 3D volume was then randomly rotated and their brightness was randomly altered. Random blurring was applied using a Gaussian filtre of a randomly selected standard deviation. The order of the images used during training was also randomly determined. Therefore, across the training epochs, the CNN was exposed to images with different levels and patterns of augmentations. Whenever model performance is evaluated, the original CTP maps without random alteration were used, and the central 12 slices were selected for CTP maps.

The CNN was constructed as a regression model. The loss function used to train the CNN was mean squared error between the predicted mRS and ground truth labels. We added a sigmoid activation function to confine the prediction values between 0 and 1, and rescaled the ground truth labels accordingly. For example, an mRS of 1 was rescaled to 0.25, 2–0.5, 3–0.625, 4–0.75, 5–0.875 and 6–1. To update the network parameters, the Adam optimiser^31^ was used with default settings.

To prevent overfitting, early stopping was applied. During the training process, if the AUC on the validation set did not improve for 20 consecutive epochs, the training was terminated. Otherwise, models were trained for 150 epochs. The validation set was used to select the hyperparameters of the CNN model that showed the best prediction AUC. The hyperparameters include learning rate, batch size and the number of convolutional layers. The optimal learning rate, batch size and number of layers were 0.005, 6 and 5 respectively. To identify regions of CTP that contributed most strongly to the predicted mRS in the CNN, saliency mapping was performed by Grad-Cam.^32^ A random selection of 50 saliency maps were reviewed. All CNN models were run on a Nvidia A100 16GiB GPU using pytorch version 1.9.0, cuda version 11.1 and cudnn version 8.0. The computation time to analyse one CTP volume was 131.5 ± 20.0 ms on an Intel Xeon CPU and 1.6 ± 0.8 ms on a Nvidia A100 GPU. The source code is published (https://github.com/Yutong441/deepCTP). Our manuscript followed the Machine Learning Predictive Models reporting guideline.^33^

## Results

### Baseline characteristics

Two hundred thirty subjects meet the inclusion criteria for the derivation cohort and 129 for the replication cohort. Of those in the derivation cohort, 184 comprised the training set and 46 the validation set. Demographic and CTP imaging features of subjects in different cohorts are shown in Table 1.

**Table 1.** Demographic and CTP imaging features in all datasets.

	Cohort			*p* value
	Training	Validation	Replication	
Sample size	184	46	129	
Demographics and risks factors				
Sex (Females), *n*(%)	96 (52.2)	18 (39.1)	58 (45.0)	0.467
Age (Years), median (IQR)	77.0 (68.0–83.0)	73.0 (65.2–82.0)	76.0 (65.0–85.0)	0.412
Hypertension, *n*(%)	111 (60.3)	30 (65.2)	72 (55.8)	0.366
Hypercholesterolaemia, *n*(%)	48 (26.1)	11 (23.9)	43 (33.9)	0.128
Atrial fibrillation, *n*(%)	46 (25.0)	17 (37.0)	47 (36.4)	0.096
Heart failure, *n*(%)	11 (6.0)	1 (2.2)	2 (1.6)	0.154
Smoking, *n*(%)^a^	71 (38.6)	22 (47.8)	68 (52.7)	0.033
Diabetes, *n*(%)	25 (13.6)	11 (23.9)	17 (13.3)	0.653
Features of stroke				
Side of stroke (Right), *n*(%)	88 (47.8)	24 (52.2)	56 (43.4)	0.394
Pre-stroke mRS, median (IQR)	0.0 (0.0–0.0)	0.0 (0.0–0.0)	0.0 (0.0–0.0)	0.186
Baseline NIHSS, median (IQR)	12.0 (7.0–17.0)	12.7 (8.0–18.0)	9.0 (4.8–18.0)	0.014
Outcome measures				
mRS after 3 months, median (IQR)	2.0 (1.0–4.0)	2.0 (1.0–4.0)	1.0 (0.0–6.0)	0.028
Unfavourable mRS after 3 months, *n*(%)	108 (58.7)	28 (60.9)	57 (44.2)	0.009
NIHSS after 24 h, median (IQR)	5.0 (2.0–14.2)	5.0 (1.0–10.5)	3.5 (1.0–10.2)	0.119
Absence of ICH, *n*(%)^b^	159 (86.4)	37 (80.4)	123 (96.1)	0.003
Onset-to-needle time (min), median (IQR)	162.5 (120.0–200.5)	153.5 (132.8–185.0)	155.0 (114.0–202.0)	0.293
CTP parameters				
Total lesion volume (mL), median (IQR)	73.2 (42.0–134.5)	76.8 (31.1–123.7)	40.0 (12.0–104.0)	<0.001
Core volume (mL), median (IQR)	10.8 (3.8–22.9)	12.9 (5.0–28.1)	6.0 (1.0–40.0)	0.098
Penumbra volume (mL), median (IQR)	58.9 (31.2–95.6)	58.8 (19.3–89.5)	32.0 (10.0–66.0)	<0.001
Mismatch ratio (ml/mL), median (IQR)	6.8 (3.6–13.9)	5.2 (3.0–8.2)	3.7 (1.9–9.3)	<0.001

The *p* values between derivation and validation cohorts are calculated using *χ*^2^ test with Yates correction for categorical variables. For continuous variables, unpaired *t* test was used if the variable is normally distributed (Shapiro test *p* values >0.05). Wilcoxon rank sum test was used if the variable is not normally distributed.

a Smoker: current and/or previous smoking history.

b ICH: intracranial haemorrhage on 24-h NCCT.

### Features predicting outcome

**Figure 2.**
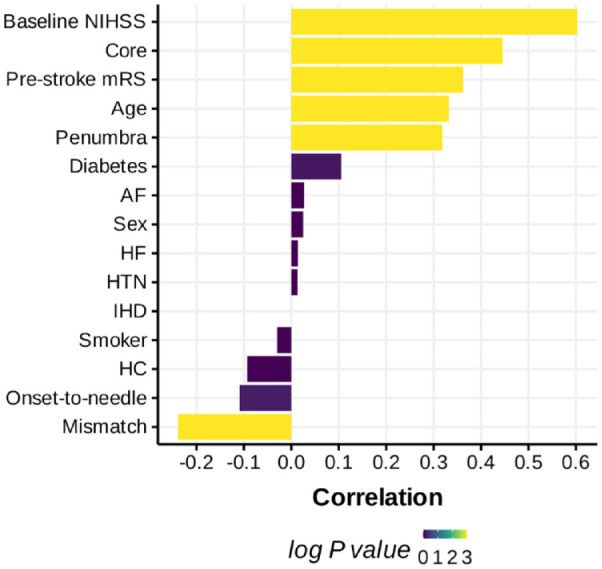
Correlation between clinical and CTP features and 3 months mRS in descending order. Correlation was performed using the Spearman test. The *p* values are adjusted for multiple testing using Bonferroni correction. *p* values were log transformed such that log *p* value of 0, 1, 2 and 3 corresponds to *p* values of 1, 0.1, 0.01 and 0.001 respectively. Abbreviations: IHD: ischaemic heart disease; AF: atrial fibrillation; HTN: hypertension; HC: hypercholesterolaemia; HF: heart failure; Core: ischaemic core volume; Smoker: current and/or previous smoking history.

Overall, baseline NIHSS, core and penumbra volumes demonstrated highest correlations with 3 months mRS, while demographic features were not correlated (Figure 2). Baseline NIHSS had the highest correlation with 3 months mRS. Higher core and penumbra volumes are associated with higher mRS, whereas a higher mismatch ratio was associated with a lower mRS.

### CNN performance versus conventional MISTAR models

In the replication dataset, the prediction AUC of 3 months mRS was 0.583 (95% CI 0.480–0.686) using the original MISTAR model, which applied pre-established thresholds on core and penumbra volumes^13^ in a decision tree model to predict 3 months mRS. Prediction was improved when using the ‘modified’ MISTAR model in which the thresholds had been obtained from the derivation dataset (AUC = 0.670, 95% CI, 0.571–0.769). The CNN model performed better (AUC = 0.792, 95% CI, 0.707–0.877) than the original (*p* < 0.001, *t* test) and modified MISTAR models (*p* < 0.001, *t* test) (Table 2).

**Table 2.** Performance of different models in predicting mRS at 3 months.

	AUC (95% CI)	Sensitivity (95% CI)	Specificity (95% CI)	Accuracy (95% CI)
MISTAR (original)	0.583 (0.480–0.686)	0.708 (0.630–0.786)	0.457 (0.371–0.543)	0.550 (0.464–0.636)
MISTAR (modified)	0.670 (0.571–0.769)	0.708 (0.630–0.786)	0.632 (0.549–0.632)	0.632 (0.549–0.715)
CNN	0.792 (0.707–0.877)	0.708 (0.630–0.786)	0.877 (0.820–0.934)	0.814 (0.747–0.881)
CNN + demo^a^	**0.865** (0.794–0.936)	**0.729** (0.652–0.806)	**0.926** (0.881–0.971)	**0.853** (0.792–0.914)

Sensitivity is with respect to unfavourable outcomes (mRS 3–6). Specificity is with respect to favourable outcomes (mRS 0–2). Bold numbers indicate the metric in the best performing model.

a CNN + demo: machine learning model combining CNN-derived features with clinical features using lasso model.

**Figure 3.**
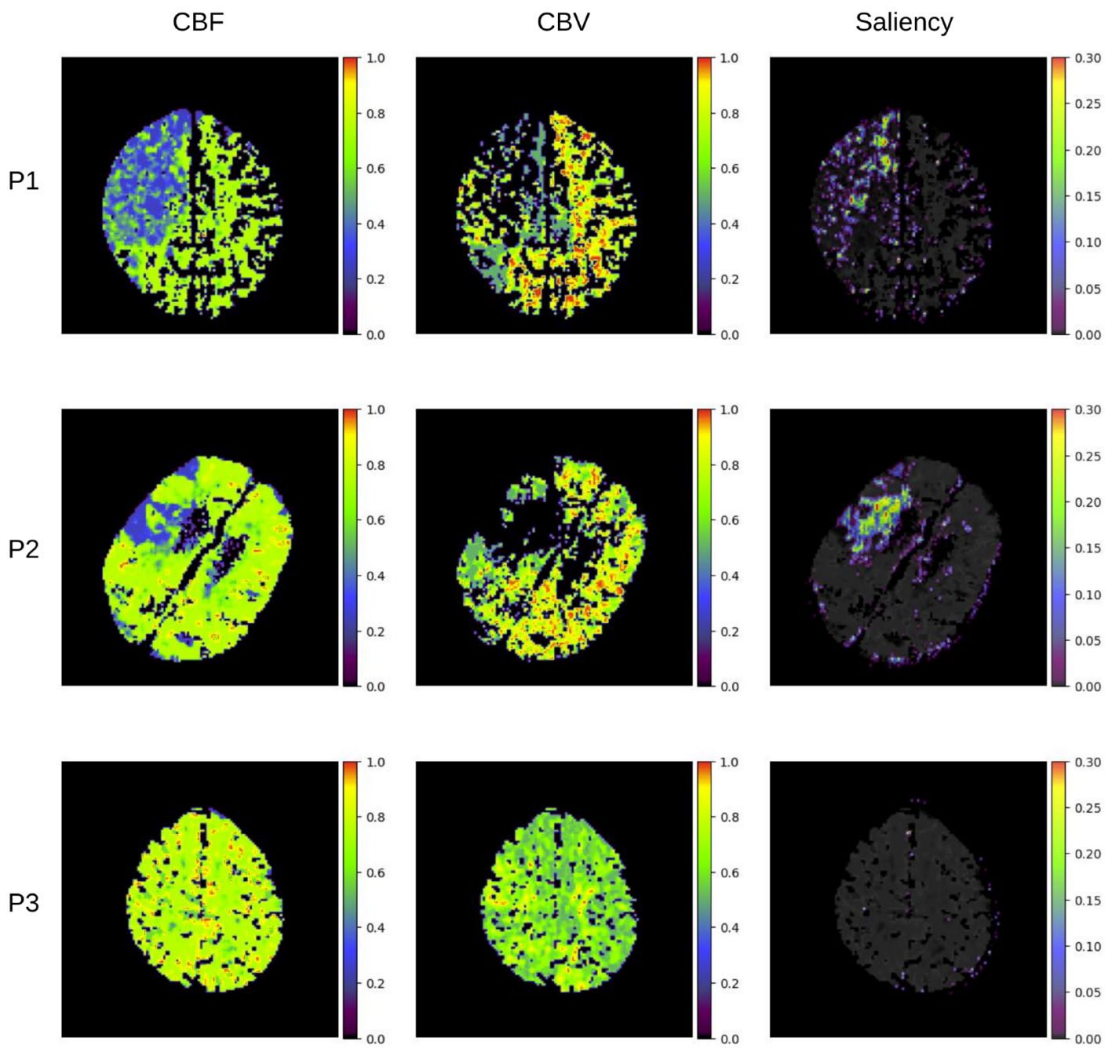
Saliency mapping of the CTP regions that most strongly activate the CNN. The cerebral blood flow (CBF), cerebral blood volume (CBV) and the saliency map (saliency) from three patients in the replication cohort were shown (P1, P2 and P3). The values in each map were rescaled to between 0 and 1 (minimum and maximum values in a volume of a CTP map respectively). In the saliency maps, regions with higher values indicate their higher degree of importance in contributing towards the CNN output.

Saliency mapping showed that the regions in the CTP maps that most strongly contribute to CNN output overlap with regions of low CBF and CBV–the ischaemic core (Figure 3 patients P1 and P2).Normal tissue regions did not contribute to CNN output (Figure 3 patient P3). This suggests the CNN model predominantly relied on data from the ischaemic core in predicting mRS at 3 months.

### Combining features

We extracted the values of the last layer of the CNN for each patient and merged them with clinical features. These features were: age, sex, hypertension status, previous ischaemic heart disease, history of heart failure, status of hypercholesterolaemia, smoking history, diabetes, pre-stroke mRS, and baseline NIHSS. The combined CNN-derived and clinical features serve as the input in various machine learning models. Lasso achieved the highest AUC in mRS prediction amongst the different machine learning models (Table 3). The AUC in mRS prediction by lasso model is significantly higher than the second highest-performing model–GPR (*p* = 0.017, *t* test). Adding in demographic features resulted in a small but statistically significant increase in the AUC to 0.865 (95% CI, 0.794–0.936) (*p* < 0.001, *t* test).

**Table 3.** Performance of different machine learning models in predicting mRS at 3 months using both CNN-derived and clinical features.

	AUC (95% CI)	Sensitivity (95% CI)	Specificity (95% CI)	Accuracy (95% CI)
KNN	0.783 (0.697–0.869)	0.583 (0.498–0.668)	0.852 (0.791–0.913)	0.752 (0.677–0.827)
SVM (Radial)	0.854 (0.781–0.927)	0.667 (0.586–0.748)	0.877 (0.820–0.934)	0.798 (0.729–0.867)
SVM (Linear)	0.836 (0.759–0.913)	**0.792** (0.722–0.862)	0.741 (0.665–0.817)	0.760 (0.686–0.834)
lasso	**0.865** (0.794–0.936)	0.729 (0.652–0.806)	**0.926** (0.881–0.971)	**0.853** (0.792–0.914)
RF	0.849 (0.774–0.924)	0.688 (0.608–0.768)	0.864 (0.805–0.923)	0.798 (0.729–0.867)
GPR	0.855 (0.782–0.928)	0.688 (0.608–0.768)	0.877 (0.820–0.934)	0.806 (0.738–0.874)

Sensitivity is with respect to unfavourable outcomes (mRS 3–6). Specificity is with respect to favourable outcomes (mRS 0–2). Bold numbers indicate the metric in the best performing model.

KNN: k-nearest neighbours; SVM: support vector machine; RF: random forest; lasso: least absolute shrinkage and selection operator; GPR: Gaussian Process with radial kernel.

## Discussion

In this study we incorporated a deep neural network into CTP-based prediction of clinical outcomes in patients with acute ischaemic stroke undergoing thrombolysis. Using this approach we achieved a high accuracy of prediction of 3 months mRS, which exceeded that achieved by a conventional clinically available CTP analysis algorithm. Rapid automated analysis of CTP images has become an important part of acute stroke management, with the results of recent trials showing penumbral imaging using CT selection allows patients who may benefit from both thrombolysis outside the 4.5 h time window, and from thrombectomy. Our results demonstrate that deep neural network approaches may be useful in analysis of CTP images in the acute stroke setting.

We compared performance in predicting mRS 3 months post-stroke with that of MISTAR–a clinically available decision tree method, and showed superior performance with an AUC of 0.792 versus 0.583. As the original MISTAR model had been developed using a different derivation dataset, we developed a modified MISTAR model in which the thresholds were developed from the derivation dataset in this study. This performed better than the original MISTAR model (*p* < 0.001, *t* test), but was still outperformed by the CNN model. This demonstrates that a technique such as CNN, which analyses multiple features of the CTP maps, can achieve superior performance to one which uses derived parameters from the CTP maps. Of note the performance of the MISTAR decision tree algorithm was worse than the previously published AUC of 0.870,^13^ both in the original implementation, and in the implementation fitted to the derivation cohort. The reason why it performed less well in this setting is unclear.

We avoided using information collected 24 h after stroke onset, such as NIHSS or follow-up CT scans, unlike Brugnara et al.^14^ This is because these features are not available in acute settings for thrombolysis decision making, even though they can improve prediction significantly.^14,34^ Without using those features, our approach still reaches a similar performance as Brugnara et al. (AUC = 0.856), demonstrating the robustness of our CNN-based prediction algorithm.

Our study has a number of strengths. We included both a derivation and replication cohort. Data was collected on consecutive acute stroke patients and in the clinical as opposed to research setting, making it representative of routine clinical care. The researchers collecting outcome data were blinded to the results of the CTP analysis. Through saliency mapping, we demonstrated the success of CNN in predicting post-thrombolysis mRS may be because it was able to delineate the ischaemic core better than the threshold-based approach in commercial segmentation softwares such as MISTAR.^35^

However, it also has limitations. Given the single centre nature of the study, the dataset is relatively homogeneous. This may overestimate the accuracy of the CNN model and limit its generalisability. For example, in the study that developed the prediction model using MISTAR-derived volume measures^13^, the derivation and replication cohorts showed a wider range of age and baseline NIHSS than that of the current study. As the MISTAR model was developed to accommodate a wider cohort of stroke patients, this could explain why it performed less well in predicting thrombolysis outcome, compared with our CNN model that was developed on a less diverse cohort. However, the modified MISTAR model, which had been retrained using our cohort, showed lower performance than the CNN, suggesting the benefits of using CNN versus MISTAR-derived lesion volumes in a prediction model.

Furthermore, there was selection bias in this study. In some patients mRS at 3 months were unattainable, as they were repatriated to other hospitals, or discharged without follow-up. This may create class imbalance to our dataset if they do not have the same outcome as the included patients. For example, those repatriated to other hospitals may require further rehabilitation and include patients with worse functional outcomes. Secondly, we excluded patients with previous stroke because regions of previous infarction can lead to mislabelling of core and penumbra.^36^ Future studies need to validate the CNN performance in patients with previous stroke.

Given that our dataset mainly consists of patients within the conventional thrombolysis time window, it is uncertain whether the same prediction accuracy would apply to patients outside this window, where CTP is mainly indicated.^37^ Hence, our model is not tailored to the main population undergoing CTP and future studies are required to validate our study on those patients. It is also uncertain whether the model would apply to patients undergoing thrombectomy. It has suggested the thresholds may vary in this group compared with thrombolysis.^38^ However the same methodology could be applied to thrombectomy datasets to derive tailored predictive algorithms.

In conclusion, our results demonstrate that a CNN-based approach which utilises the CTP maps results in good prediction of outcome in acute stroke patients undergoing thrombolysis.

## Supplemental Material

sj-pdf-1-eso-10.1177_23969873231183206 – Supplemental material for Prediction of response to thrombolysis in acute stroke using neural network analysis of CT perfusion imaging Click here for additional data file. Supplemental material, sj-pdf-1-eso-10.1177_23969873231183206 for Prediction of response to thrombolysis in acute stroke using neural network analysis of CT perfusion imaging by Yutong Chen, Daniel J Tozer, Weiran Liu, Edward J Peake and Hugh S Markus in European Stroke Journal

## References

[1] Emberson J Lees KR Lyden P , et al. Effect of treatment delay, age, and stroke severity on the effects of intravenous thrombolysis with alteplase for acute ischaemic stroke: a meta-analysis of individual patient data from randomised trials. Lancet 2014; 384: 1929–1935. 25106063 10.1016/S0140-6736(14)60584-5 PMC4441266

[2] Goyal M Menon BK van Zwam WH , et al. Endovascular thrombectomy after large-vessel ischaemic stroke: a meta-analysis of individual patient data from five randomised trials. Lancet 2016; 387: 1723–1731. 26898852 10.1016/S0140-6736(16)00163-X

[3] Ermine CM Bivard A Parsons MW , et al. The ischemic penumbra: from concept to reality. Int J Stroke 2021; 16: 497–509. 33818215 10.1177/1747493020975229

[4] Lin L Bivard A Parsons MW . Perfusion patterns of ischemic stroke on computed tomography perfusion. J Stroke 2013; 15: 164–173. 24396810 10.5853/jos.2013.15.3.164 PMC3859000

[5] Thomalla G Simonsen CZ Boutitie F , et al. MRI-Guided thrombolysis for stroke with unknown time of onset. N Engl J Med 2018; 379: 611–622. 29766770 10.1056/NEJMoa1804355

[6] Nogueira RG Jadhav AP Haussen DC , et al. Thrombectomy 6 to 24 hours after stroke with a mismatch between deficit and infarct. N Engl J Med 2018; 54: 583–584. 10.1056/NEJMoa1706442 29129157

[7] Albers GW Marks MP Kemp S , et al. Thrombectomy for stroke at 6 to 16 hours with selection by perfusion imaging. N Engl J Med 2018; 378: 708–718 10.1056/NEJMoa1713973 PMC6590673 29364767

[8] Bivard A Spratt N Miteff F , et al. Tissue is more important than time in stroke patients being assessed for thrombolysis. Front Neurol 2018; 9: 41. 29467716 10.3389/fneur.2018.00041 PMC5808281

[9] Kidwell CS Wintermark M . The role of CT and MRI in the emergency evaluation of persons with suspected stroke. Curr Neurol Neurosci Rep 2010; 10: 21–28. 20425222 10.1007/s11910-009-0075-9

[10] Campbell BC Christensen S Levi CR , et al. Cerebral blood flow is the optimal CT perfusion parameter for assessing infarct core. Stroke 2011; 42: 3435–3440. 21980202 10.1161/STROKEAHA.111.618355

[11] Wintermark M Flanders AE Velthuis B , et al. Perfusion-CT Assessment of Infarct Core and Penumbra. Stroke 2006; 37: 979–985. 16514093 10.1161/01.STR.0000209238.61459.39

[12] Quinlan JR . Induction of decision trees. Mach Learn 1986; 1: 81–106.

[13] Bivard A Levi C Lin L , et al. Validating a predictive model of acute advanced imaging biomarkers in ischemic stroke. Stroke 2017; 48: 645–650. 28104836 10.1161/STROKEAHA.116.015143

[14] Brugnara G Neuberger U Mahmutoglu MA , et al. Multimodal predictive modeling of endovascular treatment outcome for acute ischemic stroke using machine-learning. Stroke 2020; 51: 3541–3551. 33040701 10.1161/STROKEAHA.120.030287

[15] Bentley P Ganesalingam J Carlton Jones AL , et al. Prediction of stroke thrombolysis outcome using CT brain machine learning. NeuroImage Clin 2014; 4: 635–640. 24936414 10.1016/j.nicl.2014.02.003 PMC4053635

[16] Mohana JM Madhulika MS , et al. Feature extraction using convolution neural networks (CNN) and deep learning. In: 2018 3rd IEEE international conference on recent trends in electronics, information communication technology (RTEICT), 2018, pp.2319–2323. New York: IEEE

[17] van Swieten JC Koudstaal PJ Visser MC , et al. Interobserver agreement for the assessment of handicap in stroke patients. Stroke 1988; 19: 604–607. 3363593 10.1161/01.str.19.5.604

[18] Apollo Medical Imaging Technology. Apollo Medical Imaging Technology - Product, https://www.apollomit.com/products.htm (2020, accessed 11 March 2022).

[19] Lin L Bivard A Krishnamurthy V , et al. Whole-brain CT perfusion to quantify acute ischemic penumbra and Core. Radiology 2016; 279: 876–887. 26785041 10.1148/radiol.2015150319

[20] Shapiro SS Wilk MB . An analysis of variance test for normality (complete samples). Biometrika 1965; 52: 591–611.

[21] Robin X Turck N Hainard A , et al. pROC: an open-source package for R and S+ to analyze and compare ROC curves. BMC Bioinformatics 2011; 12: 8. 21414208 10.1186/1471-2105-12-77 PMC3068975

[22] Hintze J . NCCS. NCSS, LLC. Kaysville, UT, 2007. www.ncss.com

[23] Kuhn M . Building predictive models in *R* using the caret package. J Stat Softw 2008; 28: 1–26.27774042

[24] Cunningham P Delany SJ . k-Nearest Neighbour Classifiers: 2nd Edition (with Python examples). ACM Comput Surv 2022; 54: 1–25.

[25] Hearst MA Dumais ST Osuna E , et al. Support vector machines. IEEE Intell Syst Appl 1998; 13: 18–28.

[26] Breiman L . Random forests. Mach Learn 2001; 45: 5–32.

[27] Tibshirani R . Regression shrinkage and selection via the Lasso. J R Stat Soc Ser B Methodol 1996; 58: 267–288.

[28] Rasmussen CE . Gaussian processes in machine learning. In: Bousquet O von Luxburg U Rätsch G (eds) Advanced lectures on machine learning. Berlin, Heidelberg: Springer Berlin Heidelberg, 2004; 63–71.

[29] Paszke A Gross S Massa F , et al. PyTorch: An Imperative Style, High-Performance Deep Learning Library. In: Advances in Neural Information Processing Systems. Curran Associates, Inc., https://papers.nips.cc/paper/2019/hash/bdbca288fee7f92f2bfa9f7012727740-Abstract.html (2019, accessed 28 February 2022).

[30] Wang Q Wu B Zhu P , et al. ECA-Net: efficient channel attention for deep convolutional neural networks. Epub ahead of print 7 April 2020. DOI: 10.48550/arXiv.1910.03151.

[31] Kingma DP Ba J . Adam: a method for stochastic optimization. ArXiv14126980 Cs, http://arxiv.org/abs/1412.6980 (2017, accessed 11 March 2022).

[32] Selvaraju RR Cogswell M Das A , et al. Grad-CAM: visual explanations from deep networks via gradient-based localization. Int J Comput Vis 2020; 128: 336–359.

[33] Luo W Phung D Tran T , et al. Guidelines for developing and reporting machine learning predictive models in biomedical research: a multidisciplinary view. J Med Internet Res 2016; 18: e323. 27986644 10.2196/jmir.5870 PMC5238707

[34] Monteiro M Fonseca AC Freitas AT , et al. Using machine learning to improve the prediction of functional outcome in ischemic stroke patients. IEEE/ACM Trans Comput Biol Bioinform 2018; 15: 1953–1959. 29994736 10.1109/TCBB.2018.2811471

[35] Bivard A Levi C Spratt N , et al. Perfusion CT in acute stroke: A comprehensive analysis of infarct and penumbra. Radiology 2013; 267: 543–550. 23264345 10.1148/radiol.12120971

[36] Lui YW Tang ER Allmendinger AM , et al. Evaluation of CT perfusion in the setting of cerebral ischemia: patterns and pitfalls. AJNR Am J Neuroradiol 2010; 31: 1552–1563. 20190208 10.3174/ajnr.A2026 PMC7965002

[37] NICE. Impact on NHS workforce and resources | Stroke and transient ischaemic attack in over 16s: diagnosis and initial management | Guidance | NICE, https://www.nice.org.uk/guidance/ng128/resources/impact-on-nhs-workforce-and-resources-6842988829 (2022, accessed 4 May 2023). 31211538

[38] Bivard A Kleinig T Miteff F , et al. Ischemic core thresholds change with time to reperfusion: A case control study. Ann. Neurol 2017; 82: 995–1003. 29205466 10.1002/ana.25109 PMC6712948

