# Supplementary material for: Prediction of response to thrombolysis in acute stroke using neural network analysis of CT perfusion imaging
Source: Eur Stroke J. 2023 Jun 23;8(3):629–37. doi: 10.1177/23969873231183206 (PMC10472959; doi:10.1177/23969873231183206)
Supplement: sj-pdf-1-eso-10.1177_23969873231183206 – Supplemental material for Prediction of response to thrombolysis in acute stroke using neural network analysis of CT perfusion imaging [file sj-pdf-1-eso-10.1177_23969873231183206.pdf]

# Prediction of response to thrombolysis in acute stroke using neural network analysis of CT perfusion imaging

Yutong Chen, BA[1], Daniel Tozer, PhD[1], Weiran Liu, MBBS, MA[1], Edward Peake, Bsc, Msc[2], and Hugh S Markus, DM, FMed Sc[1]

[1]Stroke Research Group, Department of Clinical Neurosciences, University of Cambridge, Cambridge, UK

[2]Department of Radiology, Addenbrookes Hospital, Cambridge, UK

## 1. SUPPLEMENTAL DISCUSSION

### 1.1 Impact of downsampling

We investigated the impact of training and testing the CNN model using full resolution CTP images (in-plane resolution being 323×323) versus downsampled images (128×128). The results show that in the validation subset of the derivation cohort, the prediction accuracies between the two approaches were the same (Supplemental Table 1). However, in the derivation cohort, the downsampling approach displayed a higher performance ($p<0.001$, t test).

One explanation is that because this study only has a moderate dataset, increasing the resolution of the CTP data increases the risk of the CNN overfitting to minor image features in the training data. Secondly, CTP data is noisy. Spatial smoothing is usually applied during data preprocessing.[1,2] This means that processed images that served as inputs to the CNN have a lower spatial resolution. Thus, we argue that downsampling CTP images from 323 × 323 voxels to 128 × 128 voxels has the benefit of reducing overfitting without sacrificing the spatial resolution to a large extent.

| Cohort | Resolution | AUC | Sensitivity | Specificity | Accuracy |
|---|---|---|---|---|---|
| Validation | 128 × 128 | 0.801 (0.669-0.933) | 0.762 (0.639-0.885) | 0.840 (0.734-0.946) | 0.804 (0.689-0.919) |
| Replication | 128 × 128 | 0.792 (0.707-0.877) | 0.708 (0.630-0.786) | 0.877 (0.820-0.934) | 0.814 (0.747-0.881) |
| Validation | 323 × 323 | 0.801 (0.669-0.933) | 0.762 (0.639-0.885) | 0.840 (0.734-0.946) | 0.804 (0.689-0.919) |
| Replication | 323 × 323 | 0.731 (0.638-0.824) | 0.771 (0.698-0.844) | 0.691 (0.611-0.771) | 0.721 (0.644-0.798) |

**Supplemental Table 1**. Impact of downsampling on performance of CNN in predicting mRS 3 months after stroke in the replication cohort and the validation subset of the derivation cohort. Brackets contain the 95% confidence interval.

### 1.2 Cohort differences

The derivation cohort is associated on average with a larger lesion volume compared with the replication cohort. In the derivation cohort, the median NIHSS at baseline is higher ($p=0.014$, Wilcoxon rank sum test) and the mRS 3 months after stroke is higher ($p=0.028$, Wilcoxon rank sum test). These differences suggest that the cases in the derivation cohort are associated with more severe stroke. Despite the discrepancy, we found no difference in the performance of the model on the derivation versus replication datasets in terms of AUC ($p=0.203$, t test) (Supplemental Table 1).

## 2. SUPPLEMENTAL REFERENCES

[1] A. M. Mendrik, E.-j. Vonken, B. van Ginneken, H. W. de Jong, A. Riordan, T. van Seeters, E. J. Smit, M. A. Viergever, and M. Prokop, “TIPS bilateral noise reduction in 4D CT perfusion scans produces high-quality cerebral blood flow maps,” *Physics in Medicine and Biology*, vol. 56, pp. 3857–3872, July 2011.

[2] A. Fieselmann, M. Kowarschik, A. Ganguly, J. Hornegger, and R. Fahrig, “Deconvolution-Based CT and MR Brain Perfusion Measurement: Theoretical Model Revisited and Practical Implementation Details,” *International Journal of Biomedical Imaging*, vol. 2011, p. e467563, Aug. 2011. Publisher: Hindawi.
